# Detection of circulating *KRAS* mutant DNA in extracellular vesicles using droplet digital PCR in patients with colon cancer

**DOI:** 10.3389/fonc.2022.1067210

**Published:** 2022-12-15

**Authors:** Jeesoo Choi, Ho Yeon Cho, Jeongseok Jeon, Kyung-A Kim, Yoon Dae Han, Joong Bae Ahn, Inbal Wortzel, David Lyden, Han Sang Kim

**Affiliations:** ^1^ Division of Medical Oncology, Department of Internal Medicine, Graduate School of Medical Science, Brain Korea 21 Project, Yonsei University College of Medicine, Seoul, South Korea; ^2^ Yonsei University College of Medicine, Seoul, South Korea; ^3^ Department of Surgery, Yonsei University College of Medicine, Seoul, South Korea; ^4^ Yonsei Cancer Center, Division of Medical Oncology, Department of Internal Medicine, Yonsei University College of Medicine, Seoul, South Korea; ^5^ Children’s Cancer and Blood Foundation Laboratories, Departments of Pediatrics, and Cell and Developmental Biology, Drukier Institute for Children’s Health, Meyer Cancer Center, Weill Cornell Medicine, New York, NY, United States

**Keywords:** cancer, colon cancer, liquid biopsy, ddPCR, extracellular vesicle, exosome, exosomal DNA, cell-free DNA

## Abstract

**Background:**

Extracellular vesicles secreted by tumor cells contain double-stranded DNA called extracellular vesicle DNA (evDNA). EvDNA is genomic DNA that reflects cancer driver mutations. However, the significance of evDNA analysis in the diagnosis and surveillance of colon cancer remains unclear. This study aimed to investigate the clinical utility of extracellular vesicles and evDNA isolated from the plasma of colon cancer patients harboring *KRAS* G12D and G13D mutations.

**Methods:**

Cell-free DNA (cfDNA) and evDNA were collected from the plasma of 30 patients with colon cancer. *KRAS* mutation status (G12D and G13D) was detected using a droplet digital polymerase chain reaction assay (ddPCR). Sensitivity and specificity were evaluated in patients with wild-type *KRAS* tumors. Mutation status was correlated with carcinoembryonic antigen (CEA) levels and overall survival (OS).

**Results:**

Thirty cfDNA and evDNA pairs showed a *KRAS* fractional abundance (FA) ranging from 0 to 45.26% and 0 to 83.81%, respectively. When compared with eight wild-type *KRAS* samples, cfDNA exhibited 70% sensitivity and 100% specificity, whereas evDNA achieved 76.67% sensitivity and 100% specificity. The concentration of evDNA was significantly lower than that of cfDNA, but it obtained a higher FA than cfDNA, while showing a positive correlation with CEA.

**Conclusions:**

Our findings demonstrate the feasibility of evDNA as a complementary tool to aid current methods of patient evaluation in the diagnosis and surveillance of colon cancer.

## Introduction

Liquid biopsy is a noninvasive method for analysis of tumor-derived materials circulating in a patient’s body fluid, primarily blood (1). It is used in the diagnosis and surveillance of cancer by monitoring treatment response and resistance-conferring mutations (2). One of the most used sources for liquid biopsy would be nucleic acids that are shed from the tumor and circulate in the bloodstream (3). DNA fragments are especially important in the detection of cancer driver mutations and are often found in the form of cell-free DNA (cfDNA), which is located in circulating tumor cells and extracellular vesicles (EV) (4–7).

Extracellular vesicles, 50–150 nm in size, are secreted by essentially all types of cells. They contain DNA, RNA, and proteins encapsulated in a lipid bilayer that can be transferred from cell to cell as signals of intracellular communication (8–10). Their secretion is exacerbated in cancer cells by active interactions with peripheral cells in the tumor microenvironment (TME) (11). The double-stranded DNA fragments found in extracellular vesicles in the size range up to a few kb are called extracellular vesicle DNA (evDNA) and represent the whole genomic DNA, making them a valuable source for the detection of mutations (12, 13).

A representation of genomic DNA in double-stranded evDNA highlights its significance as a novel source for liquid biopsy for the detection of cancer (14–16). Unlike pieces of cfDNA that are shed from apoptotic or necrotic cancer cells, extracellular vesicles are released from actively proliferating cells and are thus expected to be used in the early detection of developing disease and probable metastasis (17, 18).

*KRAS* is an important molecular switch that regulates cell survival and proliferation. A mutation in *KRAS* results in the constitutive activation of downstream signaling pathways, thereby leading to tumorigenesis (19). An aberration in the *KRAS* gene is the most frequent type of driver mutation found in cancer, occurring in approximately 20% of all cancer cases and up to 40% of colon cancer cases (20–22). In particular, point mutations in codons 12 and 13 have been validated as critical negative predictors of response to chemotherapy (22). Therefore, determining the *KRAS* mutation status of tumors is a significant step in managing patients with colon cancer.

In this study, we aimed to investigate the clinical utility of extracellular vesicles and evDNA isolated from the plasma of colon cancer patients harboring *KRAS* G12D and G13D mutations. We compared them with cfDNA and matched clinical data to determine whether they are indeed a credible tool for the diagnosis and surveillance of colon cancer.

## Materials and methods

### Patient sample collection and preparation

A total of thirty patients with colon cancer were prospectively examined. Up to 4 mL of plasma samples were extracted from each patient for the isolation of cfDNA and evDNA. Their clinical information includes age, sex, tumor TNM stage (the eighth edition of the American Joint Committee on Cancer [AJCC] cancer staging), and *KRAS* mutational status. The study was approved by the institutional review board of Severance Hospital (4-2019-0811). To isolate cfDNA, the blood was centrifuged twice at 1900 × *g* for 15 min. For ultracentrifugation, the mixture was centrifuged at 1900 × *g* for 15 min, 500 × *g* for 10 min, and 3000 × *g* for 20 min, as previously described (23). The centrifuged plasma samples were stored at -80°C for subsequent cfDNA and extracellular vesicle isolation.

### Extracellular vesicle isolation and characterization

The extracellular vesicles were isolated from plasma by ultracentrifugation. Plasma samples were centrifuged at 12,000 × *g* for 20 min. The supernatants were centrifuged twice at 100,000 × g for 70 min. The pellets were then resuspended in 200 µL of PBS and stored at -80°C. The particle number and size distribution of the isolated extracellular vesicles were measured using a Nanosight NS300 (Malvern Panalytical, Worcestershire, UK).

### Western blot analysis

The extracellular vesicles were lysed with RIPA lysis and extraction buffer (Thermo Fisher Scientific, Waltham, MA, USA), 1X protease cocktails, and phenylmethylsulfonyl fluoride (Sigma-Aldrich, Burlington, MA, USA). Denatured proteins were mixed with NuPAGE LDS Sample Buffer (Invitrogen, Waltham, MA, USA) and β-mercaptoethanol, and then heated at 95°C for 5 min. Proteins were electrophoresed on Bolt Bis-Tris Plus gels (Invitrogen) and electroblotted onto polyvinylidene difluoride (PVDF) membranes. The membranes were blocked and incubated overnight at 4°C with the following primary antibodies: flotillin-1, CD9 (Cell Signaling Technology, Danvers, MA, USA), CD81 (Novus Biologicals, Centennial, CO, USA), and β-actin (Santa Cruz Biotechnology, Dallas, TX, USA). The membranes were then washed four times with PBS-T. Immunoblots were visualized using SuperSignal West Femto Maximum Sensitivity Substrate (Thermo Fisher Scientific) and ImageQuant LAS4000 mini (GE Healthcare, Chicago, IL, USA).

### CfDNA and evDNA extraction

cfDNA was extracted from 2 mL of plasma using a NextPrep-Mag cfDNA Automated Isolation Kit (PerkinElmer, Waltham, MA, USA). Plasma samples were incubated with the binding solution, proteinase K, and magnetic beads at 56°C. The beads were then separated on a magnetic stand, and cfDNA was eluted with an elution solution. The concentration of cfDNA was measured using the Qubit dsDNA High Sensitivity Assay Kit and a Qubit 4 Fluorometer (Invitrogen). EvDNA was extracted from the extraceullar vesicle samples using AMPure XP beads (Beckman Coulter, Brea, CA, USA). Plasma samples were incubated with lysis buffer and proteinase K. Then, they were bound with magnetic beads, polyethylene glycol, and isopropyl alcohol at 56°C. Finally, the beads were separated on a magnetic stand and evDNA was eluted with nuclease-free water. The isolated evDNA was analyzed using an Agilent High Sensitivity DNA Kit and an Agilent Bioanalyzer 2100 (Agilent Technologies, Santa Clara, CA, USA) according to the manufacturer’s instructions. The amount of cfDNA and evDNA isolated from each patient is listed in **Supplementary Table 1**.

### EvDNA pre-processing

The isolated evDNA was amplified through whole genome amplification (WGA) using the REPLI-G UltraFast Mini Kit as previously described (24), followed by nested PCR to enrich the *KRAS* region. The primer sequences for nested PCR were as follows: forward primer: 5’-AAAGGTACTGGTGGAGTATTTG-3’ and reverse primer: 5’-CCTGCACCAGTAA TATGCATA-3’, respectively. Thermal cycling was performed using a SimpliAmp thermal cycler (Thermo Fisher Scientific). The following PCR conditions were used: an initial cycle at 95°C for 120 s, followed by 30 cycles of 95°C for 15 s and 60°C for 30 s, with a final cycle of DNA melting from 60°C to 95°C at a ramping rate of 0.2°C/s.

The 10 µL of Dynabeads M-270 Streptavidin (Invitrogen) was washed three times with 1X binding/washing buffer (5 mmol/L Tris-HCl, pH 7.5, 0.5 mmol/L EDTA, and 1.0 mol/L NaCl) and resuspended in 40 µL of 2X binding/washing buffer. The hybridization mixture (80 µL) was captured by mixing 10 µL of processed Dynabeads and incubating the mixture on a shaker for 30 min at room temperature. The beads were washed three times with 1X binding and washing buffer supplemented with 0.05% Tween-20 and twice with 1X binding and washing buffer only. Finally, the beads were resuspended in 20 µL water, denatured at 95°C for 2 min, and immediately placed on DynaMag magnets (Invitrogen). The suspension was then recovered for further analysis.

### Droplet digital PCR

A ddPCR was designed to recognize specific mutations in codons 12 and 13 of the *KRAS* gene (e.g., G12D, G13D), which account for the majority of *KRAS* mutations found in colon cancer. This assay was performed on QX200 Droplet Digital PCR System (BioRad, Hercules, CA, USA). The oil droplets containing up to 66 ng of cfDNA or evDNA were generated using Droplet Generation 8 (DG8) Cartridge and Droplet Generator. The generated droplets went through a PCR reaction under the following conditions: an initial cycle at 95°C for 10 min, followed by 40 cycles at 94°C for 30 s and 55°C for 1 min, and a final cycle of 98°C for 10 min and 4°C for 4 min. The droplets were analyzed in the QX200 droplet reader. The interpretation of the results was performed under the Rare Mutation Detection Best Practice Guidelines provided by Bio-Rad Laboratories. The fractional abundance (FA) was calculated as follows:


$$\text{FA}=\frac{\text{Absolute quantification of mutant alone}}{\left(\text{Absolute quantification of mutant }+\;\text{Wild-type clones}\right)}$$

Positivity was determined using a threshold set to more than 10000 total droplets, five or more positive droplets, or FA of at least 0.1%.

### Statistical analysis

Normality and lognormality were assessed using the D’Agostino & Pearson test, Shapiro-Wilk test, and Kolmogorov-Smirnov test. Analysis of paired samples of cfDNA and evDNA was performed using the Wilcoxon matched-pairs signed-rank test. The Mann–Whitney U test was used to assess the association between unpaired samples. Statistical significance was set at p< 0.05. Statistical analyses were performed using the GraphPad Prism software (version 8.0). Survival curves were generated using the Kaplan-Meier method and compared using a log-rank test. Survival curves were generated using the R statistical software version 4.2.0.

## Results

### Patient characteristics

Patient characteristics are summarized in **Table 1**. A total of 30 patients were included in the study. Blood samples of the patients were extracted within 30 days at the time of the first chemotherapy. Their median age was 60 years (range: 43 – 88 years). As for staging, 12.5% (n = 4) of patients were classified as TNM stage I, 18.8% (n = 5) as stage II, 15.6% (n = 5) as stage III, and 53.1% (n = 16) as stage IV. More than half of the patients (n = 19) had a *KRAS* G12D mutation, and the rest (n = 11) had a *KRAS* G13D mutation.

**Table 1:** Patient characteristics (n = 30).

Characteristic	n = 30	(%)
Median age (range) - yr	60	(43-88)
Male sex - no. (%)	20	(67)
Tumor stage - no. (%)		
T1	2	(7)
T2	5	(17)
T3	17	(56)
T4	6	(20)
Nodal stage - no. (%)		
N0	13	(43)
N1	6	(20)
N2	11	(37)
Stage, TNM (AJCC^1^, 8th)		
I	4	(13)
II	5	(17)
III	5	(17)
IV	16	(53)
Tumor grade or histology		
Well	2	(7)
Moderate	25	(83)
Poor	1	(3)
Mucinous or signet-ring cell	2	(7)
Microsatellite instability (MSI)		
MSI-high	3	(10)
MSS	27	(90)
*KRAS* mutation^2^		
*KRAS* G12D	19	(63)
*KRAS* G13D	11	(37)
Tumor site		
Right	10	(33)
Left	20	(67)

^1^AJCC, The American Joint Committee on Cancer.

^2^The corresponding KRAS mutation statuses were acquired by tissue biopsy.

### Characterization of extracellular vesicles, extracellular vesicle DNA, and cell-free DNA

**Figure 1:**
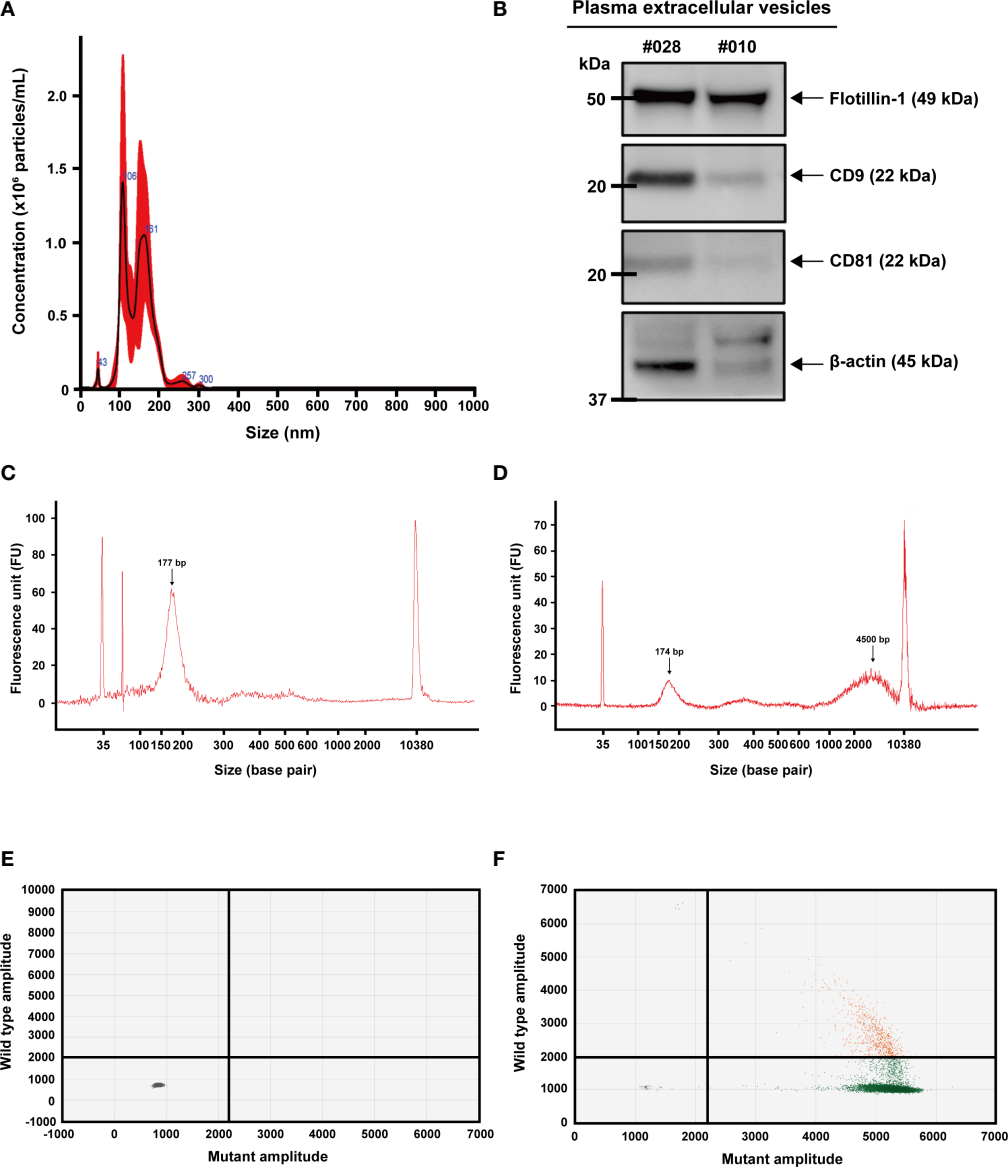
Characterization of extracellular vesicles, extracellular vesicle DNA, and cell-free DNA. **(A)** Nanoparticle tracking analysis (NTA) for counting particle number and size distribution of extracellular vesicles isolated from plasma using ultracentrifugation. **(B)** Detection of extracellular vesicle proteins by western blot analysis. Common markers (CD-9, CD-81, and Flotilin-1) were detected in extracellular vesicles isolated from plasma. **(C)** Detection of cell-free DNA (arrow) by Agilent 2100 Bioanalyzer. **(D)** Detection of extracellular vesicle DNA (right arrow) and a sign of minimally remaining cell-free DNA (left arrow). **(E, F)** 2D intensity scatter plot of *KRAS* wild-type and *KRAS* G13D mutant droplets in droplet digital PCR. Plots in each region represent droplets containing wild-type (green; lower right), mutant (blue; upper left), wild-type and mutant (orange; upper right), and no template (gray; lower left).

All extracellular vesicle samples used in this study were isolated by differential ultracentrifugation. Nanoparticle tracking analysis (NTA) was used to measure the size of extracellular vesicles isolated from the plasma of patients with colon cancer, with a size distribution of 50 to 150 nm. (**Figure 1A**). Their common protein markers, such as CD9, CD81, and Flotillin-1, were identified in the samples harvested from the two patients by western blot analysis (**Figure 1B**). Cell-free DNA (cfDNA) isolated from plasma and extracellular vesicle DNA (evDNA) extracted from extracellular vesicles were analyzed using the Bioanalyzer 2100 system. CfDNA fragments were enriched at an average size of 177 bp (**Figure 1C**), whereas evDNA fragments were enriched at an average size of 4,500 bp (**Figure 1D**). 2D intensity scatter plots generated by droplet digital PCR (ddPCR) analysis of wild-type *KRAS* (**Figure 1E**) and *KRAS* G13D mutant (**Figure 1F**) showed distinguishable scatter patterns.

### Comparing the mutation detection rates of cell-free DNA and extracellular vesicle DNA

**Figure 2:**
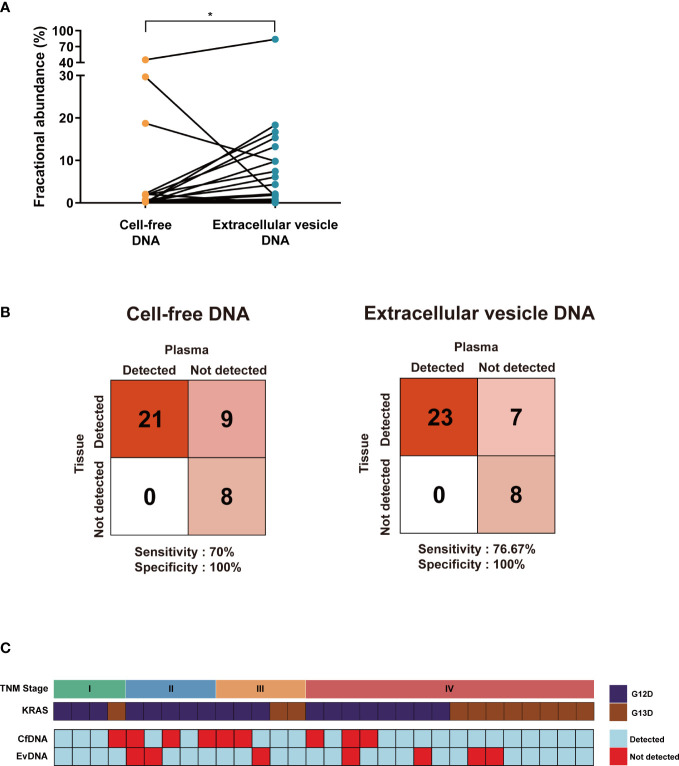
Comparing the mutation detection rates of cell-free DNA and extracellular vesicle DNA. **(A)** The mean of extracellular vesicle DNA fractional abundance was relatively higher than cell-free DNA. *P < 0.05. **(B)** A confusion matrix for detecting *KRAS* mutation of cell-free DNA (left) and extracellular vesicle DNA (right) compared with *KRAS* wild-type patient samples. The number of samples identified is noted in each box. **(C)** A detection table of cell-free DNA and extracellular vesicle DNA aligned with TNM stage and *KRAS* mutation status. Samples with fractional abundance greater than 0.1% are considered detected.

CfDNA and evDNA isolated from 30 blood samples of colon cancer patients with *KRAS* mutations were profiled using ddPCR. CfDNA yielded a median *KRAS* mutant fractional abundance (FA) of 0.3% ranging from 0 to 45.26%, while evDNA yielded a median FA of 0.78% ranging from 0 to 83.81%. When paired, the mean of evDNA FA (5.17%) was significantly higher than that of cfDNA (3.57%) (*P* = 0.0408, Wilcoxon matched-pairs signed-rank test) (**Figure 2A**). A value of FA greater than or equal to 0.1% was considered a detection. When compared with eight additional plasma samples from patients with wild-type *KRAS* and their tissue biopsy results, the *KRAS* detection rate of cfDNA showed 70% sensitivity and 100% specificity, whereas evDNA achieved a higher detection rate of 76.67% sensitivity and 100% specificity (**Figure 2B**). We then compared FAs with the TNM stage and *KRAS* mutation status of patients (**Figure 2C**). Among the 28 out of 30 (93%) samples that yielded a detection, 16 samples (53%) were detected in both types, while 12 samples (40%) were detected in only one of the DNA types. This suggests that evDNA can be a complementary source of mutant *KRAS* detection. Furthermore, a positivity was not associated with the TNM stage or type of *KRAS* mutation (G12D or G13D).

### Representation of patients’ clinical status in cell-free DNA and extracellular vesicle DNA

**Figure 3:**
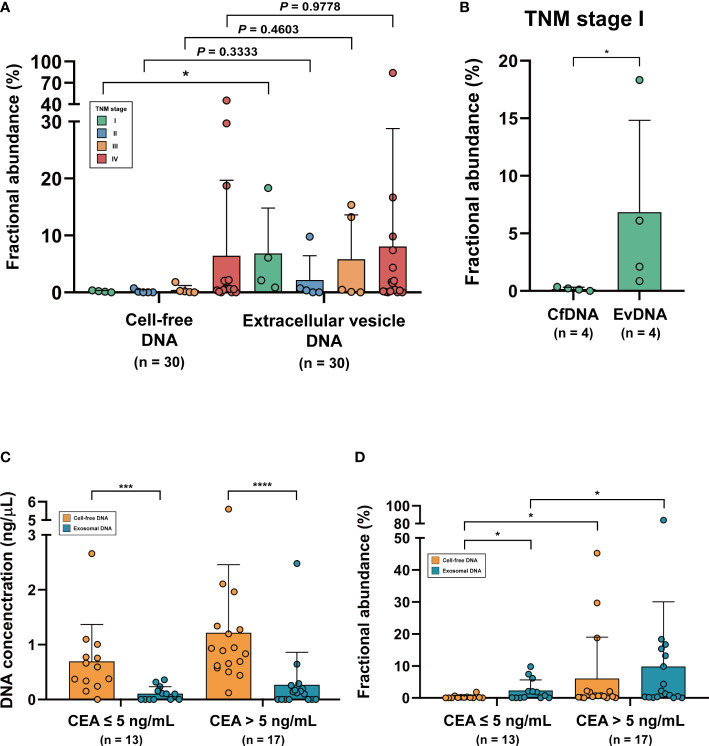
Representation of patients’ clinical status in cell-free DNA and extracellular vesicle DNA. **(A)** Fractional abundance of cell-free DNA and extracellular vesicle DNA compared with TNM stage. **(B)** Fractional abundance of cell-free DNA and extracellular vesicle DNA in TNM stage (I) *P< 0.05. **(C)** Comparison of DNA concentration of cell-free DNA and extracellular vesicle DNA in CEA-low and CEA-high groups. ***P < 0.001, ****P < 0.0001. **(D)** Comparison of fractional abundance of cell-free DNA and extracellular vesicle DNA in CEA-low and CEA-high groups. *P < 0.05.

The 30 patient samples were sorted according to TNM stage, and their FAs from cfDNA and evDNA were compared (**Figure 3A**). Mean FAs of cfDNA in each TNM stage were 0.17% (Stage I; n = 4), 0.17% (Stage II; n = 5), 0.46% (Stage III; n = 5), and 6.45% (Stage IV; n = 16), while mean FAs of evDNA were 6.84% (Stage I; n = 4), 2.17% (Stage II; n = 5), 5.83% (Stage III; n = 5), and 8.16% (Stage IV; n = 16). A significant difference was observed between cfDNA and evDNA in TNM stage I (*P* = 0.0286, Mann-Whitney U test), highlighting the detection capability of evDNA, even in the early stage of the tumor (**Figure 3B**). To more profoundly associate ddPCR profiling of *KRAS* mutations using cfDNA and evDNA with the actual clinical status of patients, we analyzed the correlation between FAs and carcinoembryonic antigen (CEA) levels. The patient cohort was divided into two groups using a cutoff value of 5 ng/mL CEA (CEA ≤ 5 ng/mL and CEA > 5 ng/mL). In both groups, the concentration of evDNA was significantly lower than that of cfDNA (*P* = 0.0005 and *P*< 0.0001, respectively, Mann-Whitney U test) (**Figure 3C**). However, FAs of evDNA were higher than cfDNA in the group with CEA less than or equal to 5 ng/mL (P = 0.0244, Mann-Whitney U test), even with lower DNA concentration (**Figure 3D**). The comparison of FA within the two groups also showed significant differences in conformity with CEA (*P* = 0.0220 and *P* = 0.0215, Mann-Whitney U test).

### Association of the fractional abundance of cell-free DNA or extracellular vesicle DNA with overall survival

**Figure 4:**
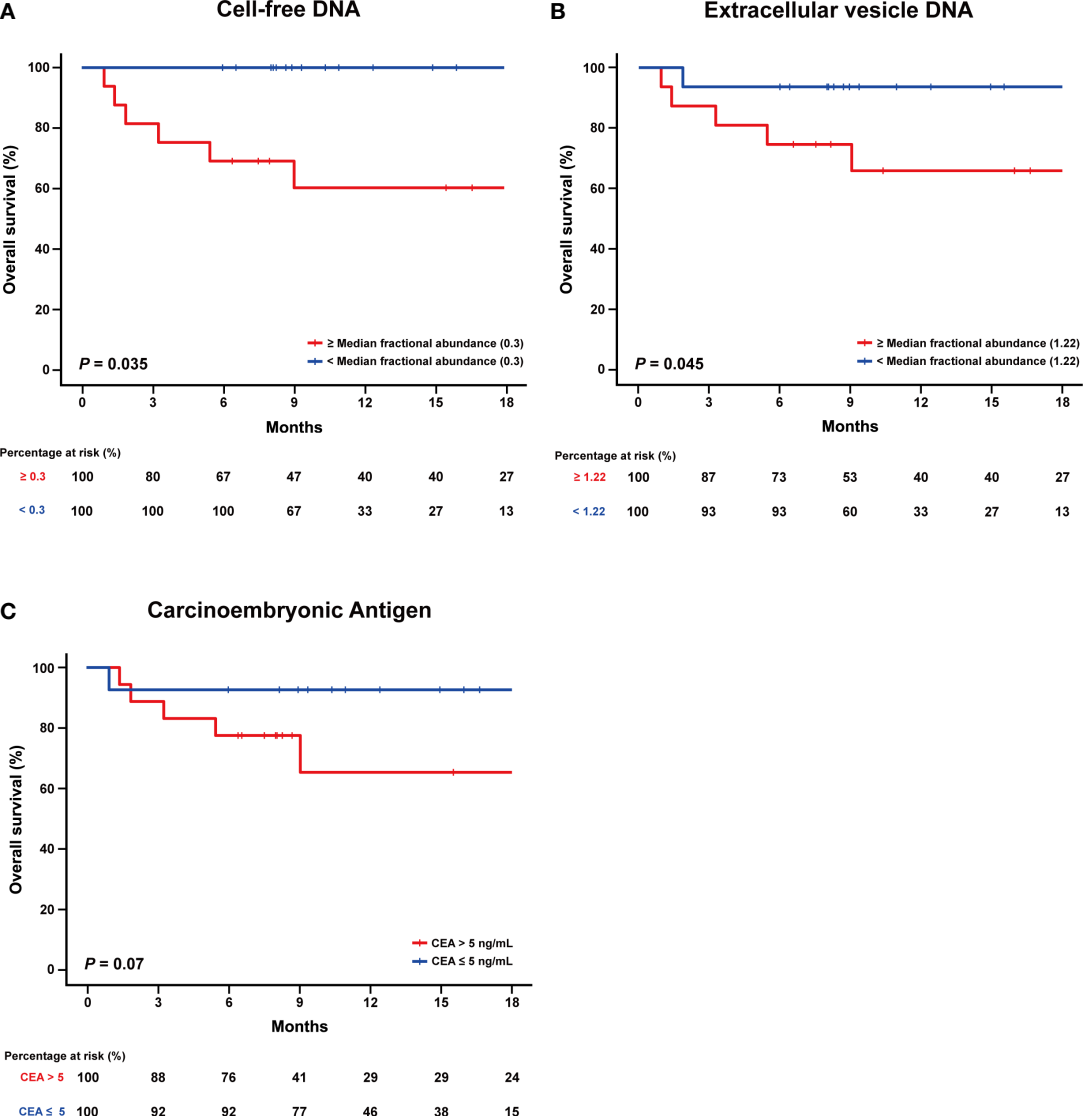
Comparing fractional abundance of cell-free DNA and extracellular vesicle DNA with overall survival (OS). **(A)** Kaplan-Meier curve of the overall survival (OS) divided according to low and high fractional abundance derived from cell-free DNA. **(B)** Kaplan-Meier curve of the overall survival (OS) divided according to low and high fractional abundance derived from extracellular vesicle DNA. **(C)** Kaplan-Meier curve of the overall survival (OS) according to CEA level.

We further evaluated whether the FAs derived from cfDNA and evDNA were associated with the overall survival (OS) of the 30 patients. The median FA of each group (0.3% and 1.2%, respectively) was set as the cutoff value to divide patients into two groups. The cutoff value for cfDNA was able to separate the two groups with significantly different overall rates (*P* = 0.035) (**Figure 4A**). For evDNA, the cutoff value was also able to separate the two groups with significantly different overall survival (*P* = 0.035) (**Figure 4B**). In contrast, CEA level correlated with OS was not able to significantly separate the patient cohort when evaluated with a cutoff value of 5 ng/mL (*P* = 0.07) (**Figure 4C**).

## Discussion

Unlike fragmented pieces of cfDNA that originate from apoptotic or necrotic cells, evDNA is safely protected in extracellular vesicles produced by actively proliferating cells (4, 8). This ensures that evDNA contains a representation of genomic DNA that wholly reflects cancer driver mutations even in the early stages of cancer development (12, 13). Thus, we hypothesized that intact evDNA would be an effective biomarker for the detection of oncogenic mutations in colon cancer. Indeed, droplet digital PCR (ddPCR) using evDNA was able to detect *KRAS* G12D and G13D mutations in colon cancer and demonstrated a comparable association with CEA and OS, which reflects the clinical status of patients. This suggests that evDNA may be valuable as an effective complementary tool for the diagnosis and surveillance of colon cancer.

In our study, an assessment of cfDNA and evDNA of colon cancer patients with *KRAS* mutations and patients with wild-type *KRAS* yielded a sensitivity of 70% and 77%, respectively. This result was consistent with that of other studies that also reported that evDNA had a higher sensitivity than cfDNA in liquid biopsies (14, 25). For instance, Krug et al. reported that evDNA (98%) yielded significantly higher sensitivity than circulating tumor DNA (82%), a tumor-specific type of cfDNA, in the detection of mutant EGFR using a targeted next-generation sequencing assay (15). Moreover, the detection rate of both cfDNA and evDNA was not affected by other patient assessment methods, such as TNM stage, suggesting that evDNA can be used regardless of the grade and stage of tumor progression.

Carcinoembryonic antigen (CEA) has an established role as a biomarker for the evaluation of colon cancer patients, and an elevation in its level is associated with metastasis and poor prognosis (26, 27). We showed that the level of FA derived from the liquid biopsy of evDNA was analogous to that of CEA, and this trend was especially highlighted in early TNM stages. In addition, the amount of evDNA acquired from the patient’s plasma was much lower than that of cfDNA. Mutant *KRAS* was detected in only one of the DNA types in approximately half of the 30 patients, suggesting that liquid biopsy using evDNA can complement the current widely used patient evaluation methods for colon cancer with a minimal amount of DNA fragments.

This study has some limitations. First, although patient-derived extracellular vesicles and extracellular vesicle DNA may serve as a source for cancer driver mutation detection, their extraction may still limit their clinical application. Currently, ultracentrifugation is known as the “gold standard” method for their isolation; however, it is a time-consuming method that requires multiple laborious steps (28). Second, the droplet digital PCR (ddPCR) method used in this study was able to detect KRAS mutants from patient-derived cfDNA and evDNA effectively, but this consistency was not observed in other types of mutations aside from *KRAS* (29, 30). Notably, ddPCR requires careful primer design and enrichment of the *KRAS* region to ensure detection. Next-generation sequencing is often suggested as a novel method to replace ddPCR for the detection of mutations, but its low accessibility and high cost still limit its application (4).

In summary, extracellular vesicle DNA from patients with colon cancer may be a novel source for the detection of cancer driver mutations. The *KRAS* mutation detection rate using evDNA was higher than that using cfDNA. It also showed consistency when compared with the conventional methods of patient evaluation. Thus, we suggest that liquid biopsy using evDNA may have a complementary role in the diagnosis and surveillance of colon cancer, as it can produce consistent results regardless of the patient’s clinical status.

## Data availability statement

The raw data supporting the conclusions of this article will be made available by the authors, without undue reservation.

## Ethics statement

The studies involving human participants were reviewed and approved by Institutional review board of Severance Hospital. The patients/participants provided their written informed consent to participate in this study.

## Author contributions

HSK conceived and designed the study. HYC, JJ, KK, and YDH collected the data. JC, HYC, JJ, and HSK performed statistical analyses. JC, HYC, and HSK wrote the manuscript. JC, HYC, KK, JBA, and HSK discussed the hypotheses and contributed to the data interpretation. DL and IW reviewed the manuscript and provided critical feedback. All authors contributed to the manuscript revision and approved the submitted version.
