# Supplementary material for: Detection of circulating KRAS mutant DNA in extracellular vesicles using droplet digital PCR in patients with colon cancer
Source: Front Oncol. 2022 Dec 15;12:1067210. doi: 10.3389/fonc.2022.1067210 (PMC9797818; doi:10.3389/fonc.2022.1067210)
Supplement: Supplementary file 1 [file Table_1.pdf]

Supplementary Table 1. Amount of cfDNA and evDNA in the study population.

Patient number (YCC-)	Cell-free DNA (ng)	Extracellular vesicle DNA (ng)
004	13.2	< 0.1*
009	27.95	0.102
010	19.68	< 0.1
016	19.84	0.102
021	20.8	< 0.1
028	< 0.1	< 0.1
029	168	2.48
030	11.13	0.112
041	11.28	0.104
046	23.16	0.232
049	28.29	0.29
052	26.73	0.258
056	32.95	< 0.1
086	35.88	0.2
089	38.22	0.152
093	30.16	0.36
104	63.18	0.12
115	7.254	< 0.1
119	22.672	< 0.1
120	24.986	< 0.1
132	4.576	< 0.1
135	10.114	< 0.1
139	17.29	< 0.1
143	18.538	< 0.1
145	17.784	0.316
146	3.64	0.14
149	15.054	0.644
151	58.85	0.17
177	40.15	< 0.1
183	79.75	0.154

* The limit of detection(LOD) is 0.1.

내과학교실/김*상/2022-11-06 18:16:13
